# Investigating gait-responsive somatosensory cueing from a wearable device to improve walking in Parkinson’s disease

**DOI:** 10.1186/s12938-023-01167-y

**Published:** 2023-11-16

**Authors:** Dongli Li, Andre Hallack, Sophie Gwilym, Dongcheng Li, Michele T. Hu, James Cantley

**Affiliations:** 1 Department of Physiology, Anatomy and Genetics, University of Oxford, Sherrington Building, Parks Road, Oxford, OX2 3PT UK; 2 Oxfordshire Neurophysiotherapy, The Bosworth Clinic, Quarry Court, Bell Lane, Cassington, OX29 4DS UK; 3 Department of Computer Science, University of Texas at Dallas, Richardson, TX 75082 USA; 4 Oxford Parkinson’s Disease Centre, University of Oxford, Oxford, UK; 5 Nuffield Department of Clinical Neurosciences, Division of Neurology, University of Oxford, Oxford, Oxfordshire UK; 6 Division of Systems Medicine, Ninewells Hospital & Medical School, University of Dundee, James Arrott Drive, Dundee, DD1 9SY UK

**Keywords:** Parkinson’s disease, Freezing of gait, Cueing, Somatosensory, Wearable, Vibration, Machine learning, Movement, Festination

## Abstract

Freezing-of-gait (FOG) and impaired walking are common features of Parkinson’s disease (PD). Provision of external stimuli (cueing) can improve gait, however, many cueing methods are simplistic, increase task loading or have limited utility in a real-world setting. Closed-loop (automated) somatosensory cueing systems have the potential to deliver personalised, discrete cues at the appropriate time, without requiring user input. Further development of cue delivery methods and FOG-detection are required to achieve this. In this feasibility study, we aimed to test if FOG-initiated vibration cues applied to the lower-leg via wearable devices can improve gait in PD, and to develop real-time FOG-detection algorithms. 17 participants with Parkinson’s disease and daily FOG were recruited. During 1 h study sessions, participants undertook 4 complex walking circuits, each with a different intervention: continuous rhythmic vibration cueing (CC), responsive cueing (RC; cues initiated by the research team in response to FOG), device worn with no cueing (NC), or no device (ND). Study sessions were grouped into 3 stages/blocks (A-C), separated by a gap of several weeks, enabling improvements to circuit design and the cueing device to be implemented. Video and onboard inertial measurement unit (IMU) data were analyzed for FOG events and gait metrics. RC significantly improved circuit completion times demonstrating improved overall performance across a range of walking activities. Step frequency was significantly enhanced by RC during stages B and C. During stage C, > 10 FOG events were recorded in 45% of participants without cueing (NC), which was significantly reduced by RC. A machine learning framework achieved 83% sensitivity and 80% specificity for FOG detection using IMU data. Together, these data support the feasibility of closed-loop cueing approaches coupling real-time FOG detection with responsive somatosensory lower-leg cueing to improve gait in PD.

**Supplementary Information:**

The online version contains supplementary material available at 10.1186/s12938-023-01167-y.

## Background

Parkinson’s disease (PD) is a progressive, neurodegenerative disorder that impairs the ability to control movement, with an estimated prevalence of 108–257/100,000 [1]. In the UK, approximately 145,000 people live with PD, a figure projected to pass 200,000 by 2035 [2]. PD is characterised by loss of dopaminergic neurones of the substantia nigra pars compacta, and by aggregation of misfolded alpha-synuclein in intracellular Lewy bodies, resulting in a number of motor pathologies including bradykinesia, rigidity, resting tremor, postural instability and gait defects including freezing-of-gait (FOG) [1]. FOG is an inability to initiate or maintain normal walking patterns, often resulting in a stochastic stop-start gait. FOG affects approximately 38% of PD patients and is associated with reduced quality of life and loss of independence [3, 4], whilst contributing to the two-fold increased risk of falling (and related injuries) with PD [5]. Current strategies to manage FOG include pharmacological interventions, physiotherapy, brain surgery (including deep brain stimulation/DBS) and cueing.

FOG transiently improves during the ‘on state’ following antiparkinsonian drug administration, yet this effect is diminished in older patients with more advanced disease [4], and FOG symptoms persist in many patients in the on state [4, 6], demonstrating that medication alone is insufficient to prevent FOG. Engagement with structured exercise programmes and balance training improves gait and stability [7, 8], although these interventions do not directly address FOG, highlighting the need for interventions targeting both postural instability and FOG [9]. Surgical interventions including bilateral deep brain stimulation (DBS) of the subthalamic nucleus can improve postural stability, gait and off-period related FOG [10–13]. However, DBS is currently used in < 5% of the PD population and alternative approaches to improve mobility more widely are urgently needed.

Cueing is defined as ‘using external temporal or spatial stimuli to facilitate gait initiation and continuation’ [14], and is endorsed by the Royal College of Occupational Therapists as a method to facilitate gait and other motor skills. Focusing attention on the external cue, rather than spontaneous walking, enables individuals to initiate and maintain gait [15, 16]. Visual, auditory and somatosensory cueing modalities have been demonstrated to improve gait in PD [17–19]. Visual cues include targets placed/projected on the floor [20, 21], displays or augmented reality devices [22, 23]. Visual cueing has been shown to improve walking speed and stride length in PD during free [22] or treadmill [21, 24] walking, whilst reducing the need for stabilising support [20]. However, visual cueing methods may distract or disturb vision, or adversely impact posture (looking downward). A systematic review of 50 published studies (involving 1892 participants) reported that rhythmic auditory cueing increases walking speed and stride length whilst reducing cadence [17]. In one study, rhythmic auditory stimulation delivered ahead of a FOG-inducing challenge showed a significant and stable reduction in number and duration of freezing episodes [25].

Although most cueing studies have used visual or auditory cues, rhythmic somatosensory cueing is a viable alternative [26]. Application of rhythmic vibration cues to the wrist during gait training programmes can increase walking speed and stride length, improve balance and reduce FOG severity [14, 27, 28]. Similarly, a small electrical stimulus can improve gait initiation [15]. Whilst the effect of cueing to improve gait is clear, the effect on overcoming FOG is inconsistent in the literature, likely due to the stochastic nature of this pathology and the large variation in study design, cue type and delivery method.

Closed-loop cueing systems couple cue delivery with a feature of the cued individual, requiring both sensor and actuator functions. Demonstrations of closed-loop systems include delivery of vibration cues to the wrist at a set point of the gait cycle (biofeedback) to reduce FOG [29, 30], coupling augmented reality visual displays to movement [23] or triggering audible cues upon deviation from a set cadence [31]. Likewise, another study used arm angle/swing to activate wrist vibration cues which improved step length, although FOG was not assessed [32]. An advanced closed-loop concept is the provision of cues in response to FOG to re-start gait: such systems have the potential to reduce task loading for the individual, representing a significant advancement over self-activated cues. Although devices capable of coupling sound cues with FOG-detection have been reported, the impact of this cueing mode on FOG-incidence and gait metrics is unclear [33, 34]. Another study delivered single-leg continuous (non-rhythmic) vibration stimulation upon FOG during guided straight line walking: this proprioceptive stimulus reduced the length of initial FOG events [35]. Key remaining challenges for closed-loop systems include improving real-time FOG detection and coupling this with effective and unobtrusive cue delivery in a real world setting [19].

In this study we aimed to investigate the feasibility of delivering rhythmic vibration cues to the lower leg in response to gait freezing, to improve gait and reduce FOG in PD patients during complex walking tasks, and to develop algorithms capable of using real-time movement data from lower leg IMU sensors to identify FOG and other gait features. This information is critical to establish closed-loop, FOG-responsive rhythmic vibration cueing systems.

## Results

### The study cohort

17 participants diagnosed with PD and self-reporting multiple daily FOG events were recruited to the study: baseline participant characteristics are presented in Table 1, and pre-study questionnaire responses summarised in Additional file 2: Table S1. Mean age was 74.5 years (range 60–84), with mean time since PD diagnosis 9.6 years (range 5–21). 18% of the cohort were female, 53% of the cohort suffered from festinating gait alongside FOG, and 29% reported a history of falling. 12 participants returned the optional FOG questionnaire (Additional file 2: Table S2), which revealed a mean FOG score of 15.1 with a range of 9–21 (0 represents no evidence of FOG; 24 represents extremely debilitating FOG preventing walking), which indicated we have captured a meaningful cross section of people with PD and FOG in our cohort. The pre-study questionnaire responses also indicate a range of activity levels, perceived triggers for FOG and techniques currently used to overcome freezing (including ‘none’, ‘internal cueing’, ‘pause and restart’ and ‘change in posture’). No serious adverse events and no falling events occurred during the study sessions.

**Table 1:** Participants’ baseline characteristics

Participant ID	Age	Gender	Years since PD diagnosis	Self-reported gait characteristics			
				Festination	Daily Freezing	Falls	FoG score
P0	75–79	Male	5–9	Yes	Yes	Yes	9.0
P1	75–79	Male	5–9	Yes	Yes		12.5
P2	75–79	Male	10–14		Yes		14.0
P3	75–79	Male	10–14	Yes	Yes		10.0
P4	75–79	Male	5–9	Yes	Yes		
P5	65–69	Male	10–14		Yes	Yes	18.0
P6	75–79	Male	10–14	Yes	Yes		
P7	70–74	Male	5–9		Yes		12.0
P8	80–84	Male	5–9	Yes	Yes		
P9	75–79	Male	10–14	Yes	Yes	Yes	21.0
P10	80–84	Male	20–24		Yes	Yes	
P11	65–69	Female	5–9		Yes		15.0
P12	80–84	Female	10–14		Yes		
P13	75–79	Female	5–9	Yes	Yes		12.0
P14	65–69	Male	15–19	Yes	Yes	Yes	21.0
P15	65–69	Male	10–14		Yes		21.0
P16	60–64	Male	5–9		Yes		15.5

### Stage A

**Fig. 1:**
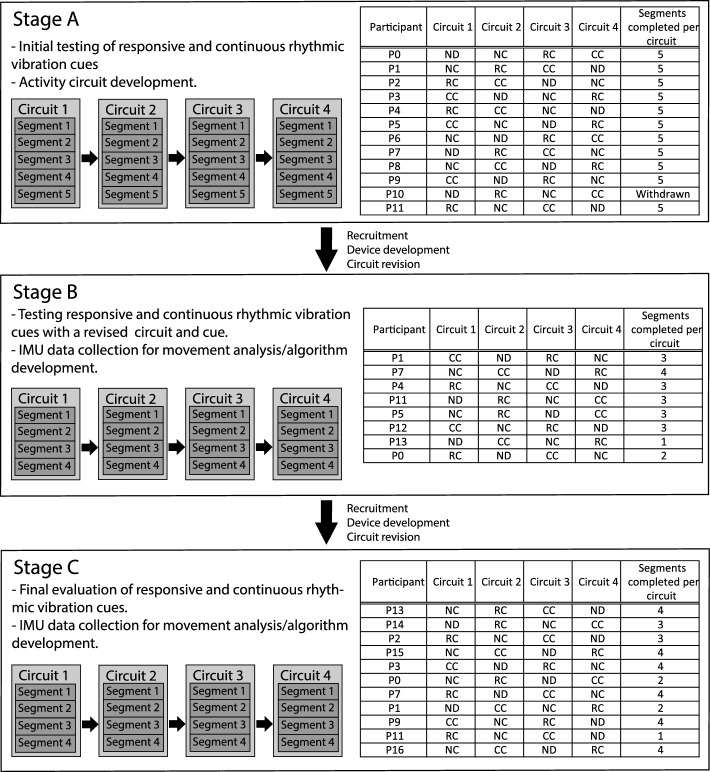
Study organisation. The study was organized in 3 stages (**A**-**C**) separated by short gaps to enable data analysis, revision of activity circuits, device servicing/improvement and participant recruitment. Each stage consisted of a series of 1 h study sessions, each involving a different participant who undertook 4 activity circuits. One of the following interventions was applied during each circuit: no device (ND); device worn, no cueing (NC); device worn, responsive (FOG-initiated) rhythmic vibration cueing (RC); device worn, continuous rhythmic vibration cueing (CC). The ordering of these interventions was systematically alternated for each participant as indicated. Each circuit consisted of a series of activity segments: the number of activity segments completed per circuit was dependent on the ability of each participant, as indicated

During stage A the functionality, safety, efficacy and comfort of rhythmic vibration cues were assessed, using a device prototyped at the University of Oxford (Additional file 2: Fig. S1; Materials and Methods). Participants undertook 4 circuits, receiving a different intervention per circuit, with the ordering of interventions systematically varied for each participant (Fig. 1). Interventions were no device (ND); device worn, no cueing (NC); device worn, responsive cueing (RC; cues initiated by the research team in response to FOG); device worn, continuous cueing (CC). Each circuit consisted of 5 activity segments (Additional file 2: Fig. S2a–e). Video data files were fragmented and viewed in random order without sound by 3 observers blinded to NC/RC/CC intervention type, but not ND: therefore, NC was used as the baseline control during pairwise analysis.

**Fig. 2:**
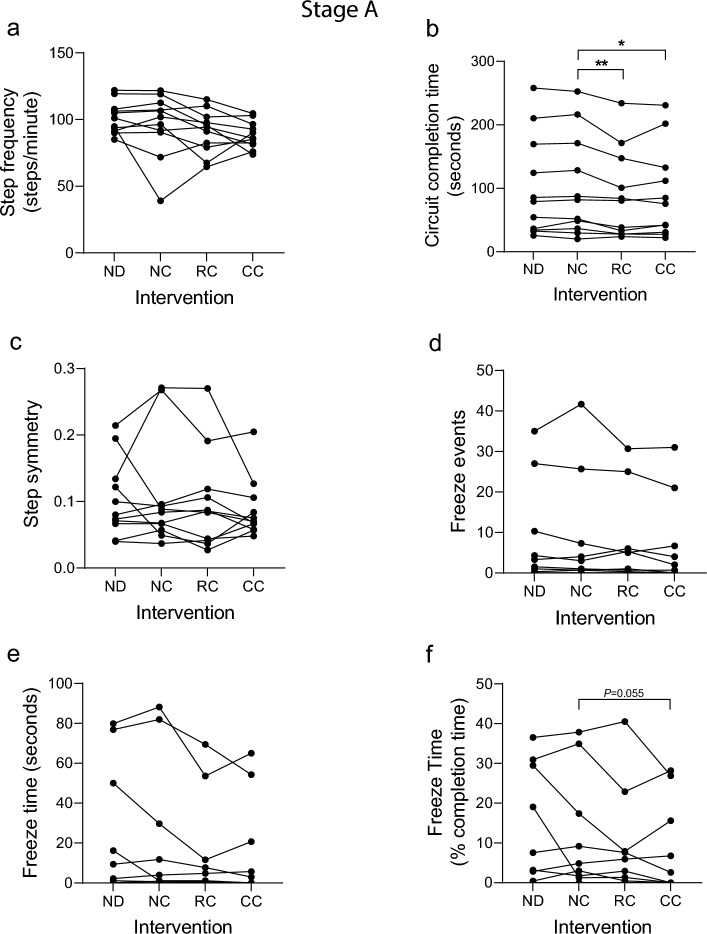
Gait metrics from analysis of Stage A video data. Participants in stage A each undertook a 1 h study session consisting of 4 activity circuits, with one of the following interventions applied during each circuit: no device (ND); device worn, no cueing (NC); device worn, responsive (FOG-initiated) rhythmic vibration cueing (RC); device worn, continuous rhythmic vibration cueing (CC). Video data were analyzed by 3 observers blinded to intervention (except ND) using GaitAnalyst software and a mean value generated for each metric during each circuit. **a** Mean step frequency (steps/min). **b** Total time to complete the circuit (seconds). **c** Step symmetry index (0 = perfect symmetry). **d** Number of freeze events detected per circuit. **e** cumulative freeze time per circuit (seconds). **f** Cumulative freeze time as a percentage of circuit completion time. Individual data points shown represent the mean response for an individual participant (*n* = 11). A Kolmogorov–Smirnov test was run to test parametric distribution. Data in (**a**, **b**) were normally distributed and analyzed using paired t-tests. Data in (**c**–**f**) were not normally distributed and were analyzed using Wilcoxon matched-pairs signed rank tests. Significant differences indicated by asterisks: *P* < 0.05*, *P* < 0.01**

12 participants attended stage A (Fig. 1). Upon commencing the study one participant (P10) was deemed to have a high fall risk and was withdrawn. The remaining 11 participants completed all 5 segments of the 4 circuits. Multiple linear regression was conducted with step frequency as the dependent variable, and participant [1–11], intervention type (1–4) and intervention circuit position (1–4) as independent variables: of these, only ‘intervention type’ contributed significantly to the model (*P* < 0.05; Table 2). Pairwise analysis of step frequency with either RC or CC, relative to the NC control, did not identify any statistically significant changes between intervention types (Fig. 2a). However, a trend for individuals with a low baseline step frequency (NC < 75 steps/min) to increase step frequency with active cueing was observed, whereas participants with a higher baseline step frequency (NC > 75 steps/min) showed a tendency to reduce step frequency with cueing (Fig. 2a), suggesting that cueing may help to regulate step frequency toward an optimal target.

**Table 2:** Step Frequency Multiple linear regression

	Fit	dF	ANOVA		*P*-Value	Coefficients (*P* values)		
	R^2^		Residual	F		Participant	Intervention type	Intervention circuit position
Stage A	0.186	3	40	3.043	0.04*	0.053	0.034*	0.466
Stage B	0.32	3	28	5.868	0.003*	0.003*	0.014*	0.927
Stage C	0.268	3	40	1.034	0.388	0.112	0.499	0.954

Multiple linear regression using step frequency as the dependent variable, and participant ID, intervention type (1–4) and intervention circuit position (1–4) as independent variables. *P < 0.05

We next quantified circuit completion time which captures aggregated walking performance throughout a range of complex activities. Pairwise analysis revealed a significant reduction in completion time with both RC and CC interventions, relative to the NC control, indicating that both forms of cueing improve walking performance (Fig. 2b). Next, we analyzed step symmetry (rhythm), which is defined as the difference in timing between alternating steps. Most participants showed step symmetry between 0 and 0.1 (with zero being perfectly symmetrical), although two participants showed very large deviations. Overall, no statistically significant effect of cueing on step symmetry was detected (Fig. 2c).

FOG events were identified from video data which, due to the cross-sectional cohort, were highly variable: two participants showed a high FOG rate (> 20 FOG events per circuit), whereas 3 participants showed no FOG. Due to the low levels of FOG recorded, no significant differences were observed in the number of freeze events (Fig. 2d) or cumulative FOG duration (Fig. 2e, f) across the cohort. However, there were several participants who showed a marked reduction in freeze time with responsive or continuous cueing.

Post-study questionnaires (Additional file 2: Table S3) revealed that 54% of participants perceived that cueing improved their walking. 75% of participants suggested that the vibration should be stronger. 2 participants suggested that vibration strength should be variable depending on the situation (‘stronger on a bad day, weaker on a good day’), 1 participant stated that cue frequency should be ‘tuned to their pace in different environments’, and another found the cue frequency to be ‘too fast when turning and too slow on the straight’. 6 participants showed either no freezing or a single FOG event, with one participant remarking that the circuits could be more challenging. All participants reported that the device was comfortable, with one stating that it was ‘very light and I didn’t notice it’ and another found the cue to be ‘comforting’ and ‘soothing’.

### Stage B

Based on participant feedback the device was modified to incorporate more powerful vibration motors (amplitude increased from 1.49G to 4.51G), and activity circuits were revised to be more challenging but with fewer segments to simplify logistics (Additional file 2: Fig. S3). Finally, the IMU device capability was activated to enable onboard collection of movement data.

**Fig. 3:**
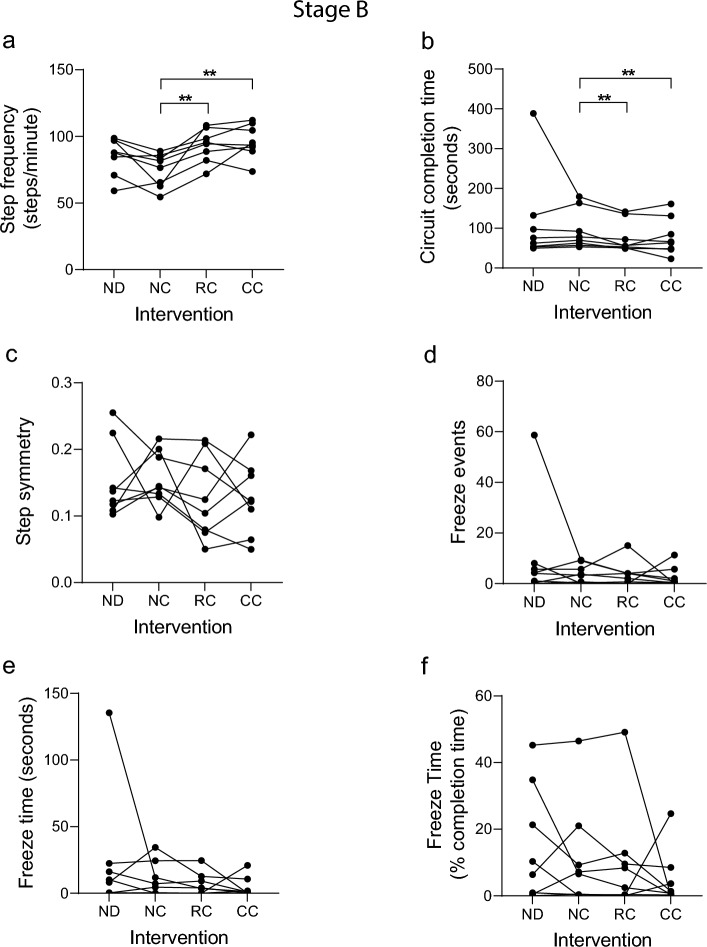
Gait metrics from analysis of Stage B video data. Participants in stage B each undertook a 1 h study session consisting of 4 activity circuits, with one of the following interventions applied during each circuit: no device (ND); device worn, no cueing (NC); device worn, responsive (FOG-initiated) rhythmic vibration cueing (RC); device worn, continuous rhythmic vibration cueing (CC). Video data were analyzed by 3 observers blinded to intervention (except ND) using GaitAnalyst software and a mean value generated for each metric during each circuit. **a** Mean step frequency (steps/min). **b** Total time to complete the circuit (seconds). **c** Step symmetry index (0 = perfect symmetry). **d** Number of freeze events detected per circuit. **e** cumulative freeze time per circuit (seconds). **f** Cumulative freeze time as a percentage of circuit completion time. Individual data points shown represent the mean response for an individual participant (*n* = 8). A Kolmogorov–Smirnov test was run to test parametric distribution. Data in (**a**) were normally distributed and analyzed using paired t-tests. Data in (**b**, **c**-**f**) were not normally distributed and were analyzed using Wilcoxon matched-pairs signed rank tests. Significant differences indicated by asterisks: *P* < 0.01**

8 participants took part in stage B with one participant completing all 4 segments of each circuit, and 7 participants completing 1–3 segments (Fig. 1), indicating that the revised circuits pose an appropriate walking challenge for our cohort. Multiple linear regression of step frequency revealed that both ‘intervention type’ and ‘participant’ contributed significantly (*P* < 0.05) to the model (Table 2). Further analysis revealed that step frequency increased significantly with both RC and CC, relative to NC (Fig. 3a). In addition, a significant reduction in circuit completion time with RC and CC, relative to NC (Fig. 3b), was observed, reproducing the positive effect recorded in stage A. Taken together, these data reflect an improvement in walking ability within the cohort when assisted with either cueing modality. Interestingly, the mean step frequency at baseline (NC) in stage B was 74 steps/min, whereas in stage A this was 96 steps/min: this is likely driven by differences in the cohort/participants and activity circuits. This may explain in part why for stage A we saw a trend for reduced step frequency in individuals with higher baseline step frequency, but an increase in step frequency for all participants in stage B where baseline step frequency was lower. The step symmetry index was higher in stage B relative to stage A, indicating worse step symmetry. There were no significant differences between the intervention types (Fig. 3c), although a non-significant trend toward improved step symmetry (reduced symmetry index) was observed in 6 participants with RC relative to NC. Quantification of FOG events showed substantial variation in the cross-sectional cohort: whilst some evidence for FOG was detected in all stage B participants, this was highly variable, with two participants showing minimal (1 or less) FOG events and three participants showed >10 FOG events. Due to this heterogeneity and low levels of FOG in most participants, no significant differences in the overall number or duration of FOG episodes were detected (Fig. 3d–f), although some participants did show a reduction with cueing.

Post-study questionnaire responses (Additional file 2: Table S4) revealed that 75% of participants perceived that cueing improved their walking, of which 2 participants found that cueing helped them concentrate. Only 25% of participants suggested the cue should be stronger: one suggested it should be ‘stronger, less diffuse and sharper’ and that ‘the cues tended to blend into each other’ whilst another stated that ‘it should be stronger on my bad side’ indicating that the ability to vary cue intensity independently on each leg could be a useful feature. One participant remarked that they ‘liked having their hands free’ during cue delivery, whilst another indicated that they would like the option of a manual trigger. All participants said that wearing the device was comfortable. One participant said that they had suffered from cramps for several days, and that the rhythmic vibration had a ‘soothing effect’. One individual commented that they thought the device should be quieter.

### Stage C

During stage C, activity circuit segment 3 was updated to increase the walking time and complexity of the circuit (Additional file 2: Fig. S4). The device software was further improved with a wider value range of configurable parameters (including vibration intensity, duration and frequency) to better personalise the cue for each individual.

**Fig. 4:**
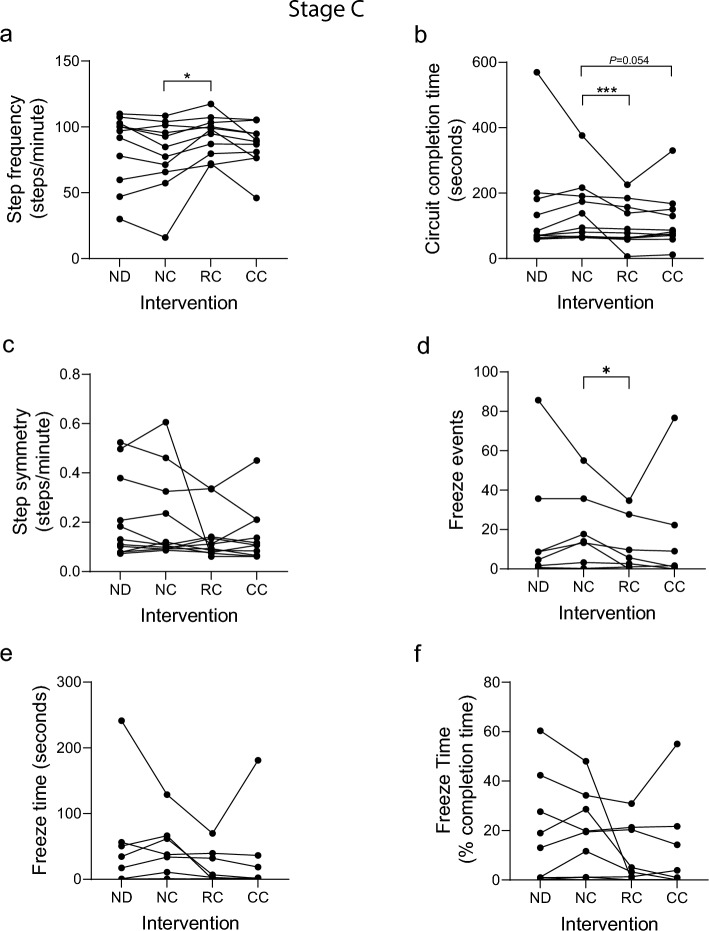
Gait metrics from analysis of Stage C video data. Participants in stage C each undertook a 1 h study session consisting of 4 activity circuits, with one of the following interventions applied during each circuit: no device (ND); device worn, no cueing (NC); device worn, responsive (FOG-initiated) rhythmic vibration cueing (RC); device worn, continuous rhythmic vibration cueing (CC). Video data were analyzed by 3 observers blinded to intervention (except ND) using GaitAnalyst software and a mean value generated for each metric during each circuit. **a** Mean step frequency (steps/min). **b** Total time to complete the circuit (seconds). **c** Step symmetry index (0 = perfect symmetry). **d** Number of freeze events detected per circuit. **e** cumulative freeze time per circuit (seconds). **f** Cumulative freeze time as a percentage of circuit completion time. Individual data points shown represent the mean response for an individual participant (*n* = 11). A Kolmogorov–Smirnov test was run to test parametric distribution. Data in (**a**) were normally distributed and analyzed using paired t-tests. Data in (**b**, **c**-**f**) were not normally distributed and were analyzed using Wilcoxon matched-pairs signed rank tests. Significant differences indicated by asterisks: *P* < 0.05*, *P* < 0.001***

11 participants took part in stage C of which 6 completed all 4 circuits (Fig. 1). In contrast to prior study stages, none of the independent variables predicted step frequency using multiple linear regression (Table 2), perhaps reflecting the high degree of variation across the cohort. However, pairwise analysis revealed a significant increase in step frequency with RC relative to the NC, but not for CC (Fig. 4a). Furthermore, a highly significant reduction in circuit completion time was identified for participants receiving RC, relative to the NC control (Fig. 4b). CC also reduced completion time, which was approaching the threshold for significance (*P* = 0.054). As in previous study stages, step symmetry was highly variable and did not show any statistically significant association with cueing, although an improvement was noted in some cued individuals (Fig. 4c). In contrast to prior study stages, 5 participants showed > 10 FOG events during baseline NC circuits (Fig. 4d), representing a higher FOG incidence. RC significantly reduced the overall number of freeze events (Fig. 4d). There was a trend for CC to reduce FOG events and freeze time, but this was not significant across the cohort (Fig. 4d–f).

**Fig. 5:**
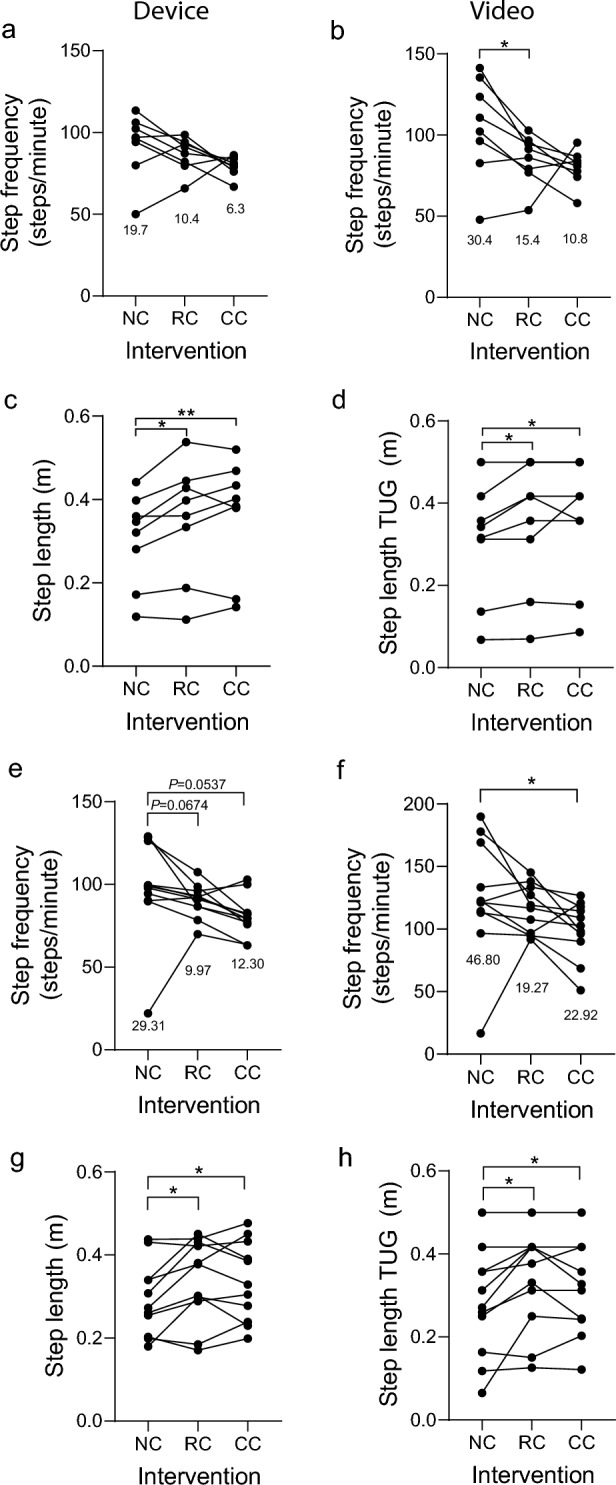
Comparison of gait analysis using IMU and video data. During stages B and C inertial measurement unit (IMU) data was collected using the GaitThaw device. Analysis of IMU data during the timed-up-and-go (TUG) test (segment 1) enabled comparison with observer analysis of video data during stage B (**a**-**d**) and stage C (**e**–**h**). Interventions analyzed were device worn, no cueing (NC); device worn, responsive (FOG-initiated) rhythmic vibration cueing (RC); device worn, continuous rhythmic vibration cueing (CC). **a** Step frequency from IMU data during stage B. **b** Step frequency from video data during stage B. **c** Step length from IMU data during stage B. **d** Step length from video data during stage B. **e** Step frequency from IMU data during stage C. **f** Step frequency from video data during stage C. **g** Step length from IMU data during stage C. **h** Step length from video data during stage C. Individual data points on graphs represent the mean response for an individual participant (**a**–**d**, *n* = 8; **e**–**h**, *n* = 11), with standard deviation indicated (a, b, e, f). A Kolmogorov–Smirnov test was used to test parametric distribution. Data in (a, b, d, f, g, h) were normally distributed and analyzed using paired t-tests. Data in (c, e) were not normally distributed and were analyzed using Wilcoxon matched-pairs signed rank tests. Significant differences indicated by asterisks: *P* < 0.05*, *P* < 0.01**

Post-study questionnaires (Additional file 2: Table S5) revealed that 91% of participants perceived that cueing improved their walking, with several participants noting it helped them to ‘slow down’ their walking pace, to ‘focus’ and ‘concentrate’ (P0, P1, P7, P11). One respondent noted that the ‘responsive cue really changed my walking: it helped me initiate walking with my right or left leg; usually I lead with my left’, and that ‘I felt more relaxed and it helped me multitask………giving me more time to judge where I’m going and better navigate corners’. The cueing was noted for preventing stride length from shortening (P14), both in straight lines and around corners, which is also captured in quantitative video and IMU data presented below (Fig. 5). All participants felt the device and cue were comfortable, although 2 noted it should be quieter. Most felt the cue intensity and frequency were appropriate, but some suggested cueing could be adjusted for different scenarios (e.g., outside vs inside).

### Detection of step frequency and step length from onboard IMU data

During study stages B and C, movement data were captured from onboard IMUs, enabling extraction of step frequency and stride length data, with verification against video data. These analyses were undertaken during the TUG segment, as this relatively simple walking task enabled clear comparisons across participants and between video and IMU data, with accurate calibration of distance travelled. The results reveal that step frequency calculated from IMU data shows very similar trends to video analysis data (Fig. 5), with 6 of the 8 participants in stage B (Fig. 5a), and 9 of the 11 participants in stage C (Fig. 5e), showing a reduction in IMU step frequency. However, the reduction in step frequency with RC did not reach statistical significance with IMU data (Fig. 5a, e), in contrast to video data (Fig. 5b, f), suggesting that IMU analysis is less sensitive. However, step length calculated using both IMU and video data was significantly enhanced by RC and CC (Fig. 5c, d, g, h).

### Development of real time FOG detection and gait analysis algorithms

A key requirement for closed-loop cueing systems is the development of accurate FOG-detection and gait analysis algorithms, fast enough to coordinate real-time cueing responses and efficient enough to run on a typical microcontroller in a battery-powered wearable device. The main limitation for speed is the fast Fourier transform (FFT), a critical step for freeze index computation. For the embedded microprocessor in the GaitThaw device (Teensy 3.6), the expected computational cycle for a 256 point FFT is 114 µs, which yields 342 µs for 3-axis IMU data. This is considerably lower than the 10–15 ms IMU acquisition cycle required to obtain walking data [34, 37]. However, since this is a sliding window problem (only one of the 256 data points is updated at each cycle), Sliding Discrete Fourier Transform [38] was employed, further reducing computation time. Runtime system evaluation revealed an acquisition rate of 82 Hz while computing gait features and performing FOG detection. The software sketch used a total of 70kB of program storage and 20kB of dynamic memory, both 7% of the available memory for Teensy 3.6. Thus, memory usage is not a constraint for the proposed framework with these device specifications.

**Fig. 6:**
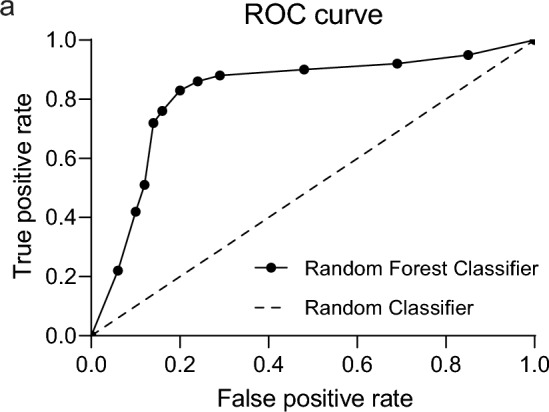
Evaluation of automated FOG-detection. We applied our FOG detection machine learning framework to IMU data collected during stages B and C, using observer analysis of video data as ground truth. The receiver operating characteristic (ROC) curve of the Random Forest Classifier for FOG detection is shown, with specificity (X-axis) plotted against sensitivity (Y-axis)

Initial testing of the proposed FOG detection framework was performed using published IMU data from the lower leg of *n* = 10 people with PD performing walking tasks in a controlled environment [34]. Importantly, these data have been manually annotated for FOG events, enabling use as a ground truth for machine learning. Leave-one-out cross-validation was used to assess the performance of our proposed framework, resulting in an average sensitivity and specificity across all participants of 91% and 89%, respectively, comparable to other machine learning experiments using this dataset [39, 40]. The FOG detection framework was next applied to IMU data from the present study (NC), which contained 694 FOG events scored by an observer. A receiver operating characteristic (ROC) curve of the Random Forest Classifier for FOG detection was generated (Fig. 6) and leave-one-out cross-validation yielded 83% sensitivity and 80% specificity for FOG detection. This is an acceptable level of accuracy, especially considering the variation in walking ability and FOG incidence in our relatively small cross-sectional cohort, which represents real world variations in FOG and gait when performing complex activities. Further improvement in FOG detection accuracy may be achieved with larger training data sets, and by clustering subjects according to walking quality/characteristics and training separate classifiers for these discrete groups.

## Discussion

In this study, we investigated the feasibility of delivering rhythmic vibration cues to the lower leg in response to gait freezing, to improve gait and reduce FOG. Our study has revealed that both responsive (transient cueing triggered remotely by the research team in response to FOG) and continuous cue delivery (continuous cueing throughout the walking circuit) can improve walking quality, as reflected in several metrics captured from video analysis data scored by blinded observers. A key metric is circuit completion time, as this captures the efficiency with which the individual navigates all activity segments posed, therefore, capturing the ability to initiate, maintain and coordinate gait throughout a range of walking, turning and other challenges. Responsive vibration cueing consistently and significantly improved circuit completion times throughout the three study stages, demonstrating the acute, positive impact of FOG-responsive (simulated closed-loop) vibration cues on freezing and walking ability throughout a complex series of tasks that reflect challenges found in the everyday environment.

Another important gait metric, and primary outcome measure for this study, is step frequency, which increased significantly across the cohort when responsive cueing was provided during stages B and C, demonstrating the impact of cue delivery. Interestingly, when analysing just the TUG segment, responsive cueing reduced the step frequency from the high baseline level recorded during the relatively unhindered straight walking of TUG. This suggests that responsive cueing helps to regulate step frequency toward the optimal target provided by the rhythmic cue. This is further reinforced by our observations during stage A where cueing had no significant impact on the higher average baseline step frequency observed during the less-challenging activity circuits, relative to stages B and C, suggesting no further gains to mean step frequency were provided by external cueing in this context. A prior study reported that delivery of continuous vibration cues from a waist mounted metronome disrupted gait relative to sound cues, and that switching cue confused some participants [41]. In the present study we saw no evidence for a disruption of gait by vibration cueing to the lower leg, but rather improvements in step frequency, stride length, circuit completion time and FOG incidence (with RC). The increase in stride length observed with both responsive and continuous cueing during free walking reproduces previous observations with visual cueing [24]. There was no evidence for a placebo effect of simply wearing an inactive device, as no significant differences between ND and NC were detected for any of the metrics analyzed.

A key strength of this study is our focus on testing the acute impact of responsive and continuous cueing on FOG, in a cross-sectional cohort. FOG is a stochastic event and attempting to capture this during a 1 h study session is challenging. Although all participants reported daily gait freezing upon entry to the study, we observed a high degree of heterogeneity during the study sessions: some participants did not freeze at all, whilst others had repeated FOG events at baseline. During stages A and B, levels of freezing at NC baseline were relatively low: during stage A, only 2 participants had > 10 freeze events and > 30 secs total freeze time; during stage B, no participants had > 10 freeze events, and 1 participant had a total freeze time of > 30 secs. Therefore, it is not surprising that cueing did not reduce the already low FOG rate during stages A and B. In contrast, participants in stage C demonstrated a higher rate of FOG: 5 individuals had > 10 FOG events and > 30secs total freeze time at NC baseline. Importantly, the acute provision of somatosensory cues in response to FOG, but not continuous cues, significantly reduced the number of FOG events during stage C, suggesting that provision of responsive cueing reduces the likelihood of future FOG events. However, the duration of total FOG events was not altered: one explanation could be that the cued individual is spending time processing how to respond to the cue [31]. Therefore, future studies may wish to incorporate additional cue-response training to test if this reduces total freeze time. The lack of a significant impact of continuous cueing on FOG events could be due to habituation.

Although many participants had minimal (1 or less) FOG events at NC baseline, and other gait metrics were variable across the cohort, the majority of participants perceived that cueing improved their walking: 50% (6 out of 12) during stage A; 75% (6 out of 8) during stage B; 91% (10 out of 11) during stage C. This increase in the perceived benefits of cueing may in part be due to the improvements made to the device between study stages, following participant feedback, which included increasing the strength and duration of vibrations. Another feature that was recommended by multiple participants is the ability to vary cue intensity and frequency on the fly: this could, for example, enable a lower frequency to be provided during tight turns, and a higher frequency during open walking. Whilst our data indicate that somatosensory cueing to the lower leg can regulate step frequency, similar to other forms of rhythmic cueing, future studies should carefully consider how cue intensity and frequency are controlled during cue delivery in different contexts, to better personalise the cue to the individual in a given environment. Finally, several participants reported that the cue was ‘comforting’, ‘soothing’ and ‘helped me to concentrate’. This reinforces the wider potential benefits of cueing to help support confidence in walking, but could also suggest that for some individuals a stronger cue may be required.

This study was designed to closely mimic challenges faced by people with PD in the real world. First, the study site selected was a ground level physiotherapy clinic, rather than a hospital setting. Second, participants navigated a series of walking/activity circuits, some including additional task loading, rather than straight line walking or use of a treadmill: this ensures that we capture cumulative gait performance across a range of different challenges. Third, we asked participants not to change their usual medication routine to capture a cross section of the PD population in their typical state. Fourth, our use of lightweight, discrete wearable cueing devices worn discretely on the lower legs, with no reported discomfort or evidence for trip hazards or gait impediment, indicate that this approach is safe and feasible in the everyday environment over longer periods of time. This was a feasibility study with a relatively small sample size. Although a positive impact on gait was identified which is encouraging, these results need to be validated in a larger study. This is particularly important given the person-to-person variation in gait metrics, FOG incidence and response to cueing observed in this study. Whether FOG-responsive or continuous somatosensory cueing can reduce fall risk was not tested in this study as we excluded participants with a high fall risk. However, the small, but significant reduction in FOG incidence, if extrapolated across a larger number of people, would be expected to reduce the risk of falling. Moreover, the regulation of step frequency observed during active cueing, along with several questionnaire comments that the cueing helped people to concentrate, could have a positive impact on fall risk [5]. Another limitation of our findings is that only 3 out of 17 participants recruited were female: although PD has a higher prevalence in men [42], and FOG has a higher prevalence in men with PD [4], this does not explain why only 18% of the cohort recruited were female: future research will need to further validate FOG-responsive cueing in women. Future studies will be required to determine the long-term efficacy of FOG-responsive vibration cueing. As cues are delivered only when required, this may reduce the potential for habituation or fatigue. Moreover, studies of continuous rhythmic cueing during gait training have demonstrated improved walking ability after a period of several weeks [27, 43]. Although posture was not assessed in the present study, prior reports have used cueing as a form of feedback to improve posture [44, 45]: Therefore, cueing in and of itself is unlikely to have a detrimental effect on posture. Finally, future research will be required to investigate the interaction between FOG-responsive cueing, antiparkinsonian medication, PD stage and other features of the disease.

A key component required for successful development of closed-loop cueing is accurate real-time detection of FOG. For example, the current DeFOG trial is testing the effect of FOG-triggered auditory cueing and instruction [46]. The accuracy of our machine learning framework was slightly lower when using IMU data from the present study compared to using the Bachlin et al. data set [34], probably due to the heterogeneity in walking ability and FOG in our cross sectional study: this does, however, provide a realistic indication of how the algorithm will behave in an unsupervised real-world setting. Further training/testing of this algorithm using IMU and video data sets gathered from larger cohorts of people with PD will be required prior to deployment in studies in the home environment where ground truth video data would not be available, and where more complex movement patterns may yield less accurate and representative features from IMU data [47]. Future developments in FOG detection that adapt to the users walking characteristics and cue responses would be an important step towards personalised cue delivery. Moreover, a tantalising prospect is identification of pre-FOG signatures from IMU data or other sensors, to pre-empt the onset of FOG and deploy responsive cueing early to maintain gait. Finally, the use of IMU data to quantify step frequency and stride length indicate this could be used to support gait monitoring in the everyday environment, providing valuable information for managing PD.

## Conclusions

This study reports improvements in gait and FOG with responsive vibration cueing in PD and provides a framework for real-time gait analysis and FOG detection for embedded devices. Together, these data demonstrate the feasibility of closed-loop cueing approaches to improve gait and reduce FOG in PD. Future development of variable cueing modalities, combined with adaptive gait analysis and FOG detection algorithms, would enable personalised closed-loop cueing for people with PD.

## Methods

### Study design

This feasibility study was conducted in accordance with the Declaration of Helsinki and was approved by the UK NHS Health Research Authority Southwest-Frenchay Research Ethics Committee (ref. 18/SW/0253). The study was sponsored by the University of Oxford, and funded by a Wellcome Trust Institutional Strategic Support Fund Translational Award (ISSF 204826/Z/16/Z). The study was retrospectively registered with clinicaltrials.gov (NCT05019469; 23/08/2021). The study sessions were conducted between 11^th^ March and 22^nd^ July 2019 at a private physiotherapy studio in Cassington, Oxfordshire (The Bosworth Clinic) under a contract with the University of Oxford. During all study sessions, participants were closely supervised and supported by a qualified neurophysiotherapist.

The study was organized into three stages, A–C, separated by gaps of a few weeks for device development (following participant feedback), revision of study activity circuits, data analysis and participant recruitment (Fig. 1). Each stage consisted of a series of 1 h study sessions, each attended by a different participant. During each study session, participants were instructed to navigate a circuit consisting of a series of walking segments (Fig. 1; Additional file 2: Fig. S2–S4), with each segment using a seated start and finish. Each participant would attempt the circuit 4 times, with one of the following interventions applied during each circuit: no device (ND); device worn, no cueing (NC); device worn, responsive (FOG-initiated) cueing (RC); device worn, continuous cueing (CC). The cue was a rhythmic vibration applied to the gastrocnemius muscle of both legs: frequency was adjusted to the approximate natural walking pace of each participant, and intensity adjusted to ensure each participant could comfortably feel the cue. Ordering of interventions was systematically alternated for each participant as they joined the study (Fig. 1), to minimise the potential for a training effect or fatigue to influence any one intervention. If a participant was judged unlikely to be able to complete all 4 circuits in full, then the number of segments per circuit was reduced. Responsive cueing consisted of delivery of vibration cues triggered remotely by the research team upon observing gait freezing, with cues stopped a few seconds after normal walking resumed, simulating closed-loop (automated) cueing. Continuous cueing was provision of left–right vibration cues throughout the entire circuit. Some participants took part in multiple study stages (A–C), some just one (Fig. 1). Participants completed questionnaires before and after each study session (Additional file 2: Tables S1–S5). The pre-study questionnaire (Additional file 2: Table S1) was designed to qualitatively assess walking ability, FOG, and strategies currently used to manage walking. Completion of an optional pre-study FOG questionnaire [48] provided a standardised FOG score (Additional file 2: Table S2). The post-study questionnaire (Additional file 2: Tables S3–S5) was designed to qualitatively assess participants’ interaction with the device and cue, their perception of their walking quality whilst being cued, and how this cueing modality could be improved.

### Participants and recruitment

Eligible participants were those diagnosed with PD, aged 18–90 years of age, who suffer from regular gait freezing (several times daily) but are able to walk unassisted for short periods. Eligible participants were recruited to the study following distribution of the study flyer to PD outpatient clinics at the John Radcliffe Hospital, local Parkinson’s UK support groups and private physiotherapy clinics in the Oxfordshire area. Eligible participants were required to be willing and able to give informed consent for participation in the study and to comply with all study requirements, as well as being fluent in English, and with a clinical diagnosis of Parkinson’s disease or idiopathic Parkinson’s disease. People that were deemed unable to participate safely in the study due to severe mental impairment, dementia or psychosis, or any other significant disease or disorder, were excluded. Other exclusion criteria included current participation in a clinical drug trial, pregnancy or breastfeeding. People with a high frequency of falls (daily), who have received DBS, or who have been diagnosed with atypical parkinsonism were excluded. We did not mandate that participants attend in the ON or OFF state, but rather asked participants to follow their usual medication routine when taking part in the study. 17 participants were recruited who all provided written informed consent.

### Design and construction of the ‘GaitThaw’ movement-tracking cueing device.

To enable delivery of vibration cues to the lower legs on demand, and the simultaneous tracking of participants’ leg movements, the non-invasive wearable ‘GaitThaw’ device was prototyped at the University of Oxford. The device consists of two 3D-printed biocompatible PLA plastic boxes (approximately the size and weight of a smartphone), which are worn around the lower leg using nylon elasticated straps either against the skin, or over an item of clothing, with the box against the gastrocnemius muscle (Additional file 2: Fig. S1a). Each box contains a printed circuit board (PCB) powered by a 3.7 V Lithium polymer battery, assembled with RoHS-compliant commercial modules including accelerometers and gyroscopes (movement tracking with 6 degrees of freedom), vibration motors for cue delivery, wireless transceivers, real-time clock, memory and basic components (Additional file 2: Fig. S1b, c). Detection of walking metrics from lower leg sensors has been reported to be more accurate than knee or waist locations [34]. The devices are linked by radio to enable coordinated delivery of vibration cues and recording of data. A third unit contains a control button which can be used by the research team to remotely configure and trigger cue delivery. Engagement of multiple vibration motors enabled cue intensity to be increased for patients with poor sensation. During device development members of the research team wore the device for many hours without any discomfort or other issues.

### Outcomes

Primary objective: to test if FOG-initiated vibration cues, provided at the lower-leg, can improve gait in PD patients. The primary outcome measure is step frequency: a statistically significant improvement of active cueing over no-cueing will indicate improved gait. Other gait quality measures include frequency of gait freeze events, duration of gait freeze events, continuous walking time, left/right rhythm, stride length, up and go time (seated to standing to walking). Secondary objective/outcome: to develop algorithms capable of identifying gait freeze events in real time using participant movement data, with > 80% accuracy.

### Video data, observer analysis and software development

All study participants consented to video recording of the study sessions. We developed custom gait analysis software coded in Python3.3 (‘GaitAnalyst’), to enable individual steps and other events/gait features to be marked and time-stamped against video recordings using keyboard strokes during video playback, which could be run at half and quarter speeds to increase accuracy of feature analysis (Additional file 2). We provide open access to the software here: https://github.com/dongchengli940126/KeyPressedTimeVideoRecord. Video recordings were fragmented into individual files for each segment (4 per circuit, 16 per 1 h session) and sound removed. Video recordings were viewed in random order and scored independently by 3 observers who had been trained to recognize the relevant gait features (all video recordings were scored by all 3 observers). Gait features scored are listed in Fig. 2–4 and the Additional file 1. During video analysis, individual keyboard strokes were used to record left and right foot strikes, providing the data to calculate step frequency (foot strikes per minute) and step symmetry (difference in timing between alternating steps with zero being perfectly symmetrical). Key strokes were also used to record the start and end of FOG events, enabling the number of FOG events and freeze time to be calculated. Each observer could recognize the no-device (ND) group, however, they were blinded with regard to the no-cue (NC), responsive cue (RC) and continuous cue (CC) interventions. Therefore, during pairwise statistical analysis, the active cueing groups (RC and CC) were compared with the device no-cue (NC) control, to avoid the potential for observer bias. The percentage coefficient of variation (%CV) between observer scores for stage A was 0.65 for step frequency, 0.14 for circuit completion time, 17.9 for step symmetry, 18.2 for freeze number, 19.7 for freeze time (secs) and 15.6 for freeze time (% completion time).

### Automated gait analysis algorithms using inertial measurement unit (IMU) data

#### Feature extraction from accelerometer data for FOG detection

First, the ‘freeze index’ was calculated, which is the ratio of the power of the freeze band (3 and 8 Hz) to the walking band (0.5 and 3 Hz) [49]. Since this ratio can be very high when there is little movement (e.g. standing still), a complementary feature, band power, was defined as the total power between 0.5 and 8 Hz. Other features that were used were variance of acceleration, L1 and L2 norms of acceleration [39] and entropy [40]. All features were computed for each accelerometer axis (*X*, *Y*, *Z*) using 256 points windows (about 2.5 s of data).

#### Machine learning for FOG detection

Supervised machine learning was performed using a random forests classifier in Python with the Scikit-Learn package. Random forest classification was selected as it is inherently fast, consisting of a sequence of binary classification trees, and is therefore better suited to real-time FOG detection than more demanding algorithms such as Support Vector Machine. As FOG detection is a highly imbalanced classification problem (i.e., the duration of normal walking greatly overwhelms that of FOG), the cost function was set to be 10 × greater for false negatives (missed FOG) than false positives (incorrect detection of FOG). The random forest was set to have a maximum size of 15, with 25 trees being trained and pruning enabled. Real-time working principles were applied during machine learning.

#### Calculation of step frequency and stride length from IMU data

Step frequency was estimated by summing steps detected and averaging over the duration of the circuit. Steps were identified by applying peak detection to gyroscopic data. Stride length was computed by extracting horizontal acceleration from IMU data, and integrating this for each pair of steps that make up the gait cycle, whilst correcting for velocity drift. This method was previously shown to generate < 10% error when estimating stride length of people with PD [50]. Both stride length and step frequency estimation methods were implemented in Matlab v.2018b (MathWorks Inc. Natick, MA, USA).

### Sample size

As this is a feasibility/pilot study, step frequency data were not available in house for use in power calculations. Sample size was, therefore, guided using power calculations performed on step frequency data reported in a prior treadmill study [26]. This indicated that a sample size of *n* = 10 would be required to detect a 25% change in step frequency (alpha 0.05). However, this is based on treadmill walking and, therefore, not representative of the complex walking circuits in the present study. Therefore, a target of *n* = 12 participants per study stage was selected.

### Statistical analysis

Study data were tested for normality using a Kolmogorov–Smirnov test (alpha = 0.05). If data were normally distributed, statistical analysis was performed using paired 2-tailed t-tests. If data failed the normality test, subsequent statistical analysis was performed using Wilcoxon matched-pairs signed rank tests. *P* < 0.05 was regarded as statistically significant. Statistical analysis and graphing were performed in Prism9 (Graphpad Software, San Diego, CA, USA). Multiple linear regression was performed in SPSS Statistics v27 (IBM, Armonk, NY, USA).

### Supplementary Information

**Additional file 1: **Study data sets.

**Additional file 2: Figure S1**. a. Image of device being worn correctly, located on gastrocnemius muscle (smart phone shown for scale only). b. Picture of internal device electronics. c. Labelling of elements depicted in b. **Figure S2**. Design of walking circuits during study stage A. a. Segment 1, timed up-and-go test. b. Segment 2, narrow restriction. c. Segment 3, passing through an open doorway. d. Segment 4, slalom around cones. e. Segemnt 5, step over and multitasking. **Figure S3**. Design of walking circuits during study stage B. a. Segment 1, timed up-and-go test. b. Segment 2, narrow restrictions. c. Segment 3, walking and turning with distraction. d. Segment 4, Move on instruction, multitasking and passing open doorway. **Figure S4**. Design of walking circuits during study stage C. a. Segment 1, timed up-and-go test. b. Segment 2, narrow restrictions. c. Segment 3, walking and tight turns with distraction. d. Segment 4, Move on instruction, multitasking and passing open doorway. **Additional file methods**: GaitAnalyst Video analysis program. **Table S1** Pre-study Questionnaire (all participants). **Table S2**. Pre-study FoG Questionnaire. **Table S3**. Post-study Questionnaire—Stage A. **Table S4**. Post-study Questionnaire—Stage B. **Table S5**. Post-study Questionnaire—Stage C.

## Data Availability

The datasets supporting the conclusions of this article are included in tabulated format as a Additional file 1. The video gait analysis software developed by the authors in Python3.3 (‘GaitAnalyst’) is provided open access in the github repository (https://github.com/dongchengli940126/KeyPressedTimeVideoRecord).

## Abbreviations

FOG: Freezing-of-gait
PD: Parkinson’s disease
CC: Continuous rhythmic vibration cueing
RC: Responsive cueing
NC: No cueing
ND: No device
IMU: Inertial measurement unit
DBS: Deep brain stimulation
TUG: Timed up-and-go test
FFT: Fast Fourier transform
ROC: Receiver operating characteristic
