# Supplementary material for: Investigating gait-responsive somatosensory cueing from a wearable device to improve walking in Parkinson’s disease
Source: Biomed Eng Online. 2023 Nov 16;22:108. doi: 10.1186/s12938-023-01167-y (PMC10652624; doi:10.1186/s12938-023-01167-y)
Supplement: Supplementary file 2 — Additional file 2: Figure S1. a. Image of device being worn correctly, located on gastrocnemius muscle (smart phone shown for scale only). b. Picture of internal device electronics. c. Labelling of elements depicted in b. Figure S2. Design of walking circuits during study stage A. a. Segment 1, timed up-and-go test. b. Segment 2, narrow restriction. c. Segment 3, passing through an open doorway. d. Segment 4, slalom around cones. e. Segemnt 5, step over and multitasking. Figure S3. Design of walking circuits during study stage B. a. Segment 1, timed up-and-go test. b. Segment 2, narrow restrictions. c. Segment 3, walking and turning with distraction. d. Segment 4, Move on instruction, multitasking and passing open doorway. Figure S4. Design of walking circuits during study stage C. a. Segment 1, timed up-and-go test. b. Segment 2, narrow restrictions. c. Segment 3, walking and tight turns with distraction. d. Segment 4, Move on instruction, multitasking and passing open doorway. Additional file methods: GaitAnalyst Video analysis program. Table S1 Pre-study Questionnaire (all participants). Table S2. Pre-study FoG Questionnaire. Table S3. Post-study Questionnaire—Stage A. Table S4. Post-study Questionnaire—Stage B. Table S5. Post-study Questionnaire—Stage C. [file 12938_2023_1167_MOESM2_ESM.pdf]

a

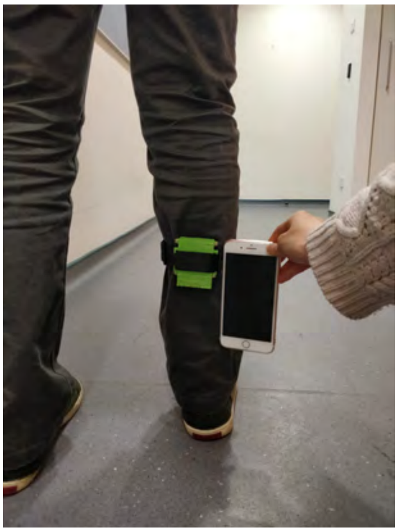

b

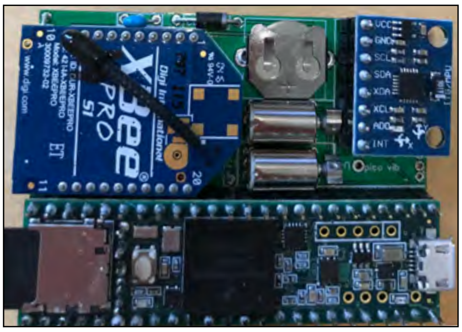

c

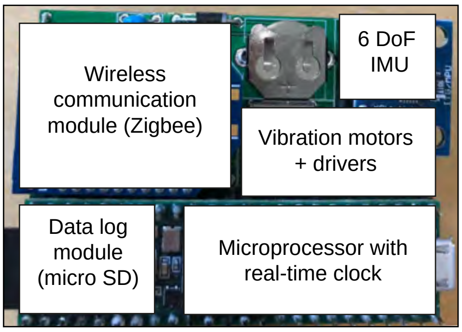

Supplementary Figure 1.

a. Image of device being worn correctly, located on gastrocnemius muscle (smart phone shown for scale only).

b. Picture of internal device electronics.
c. Labelling of elements depicted in b.

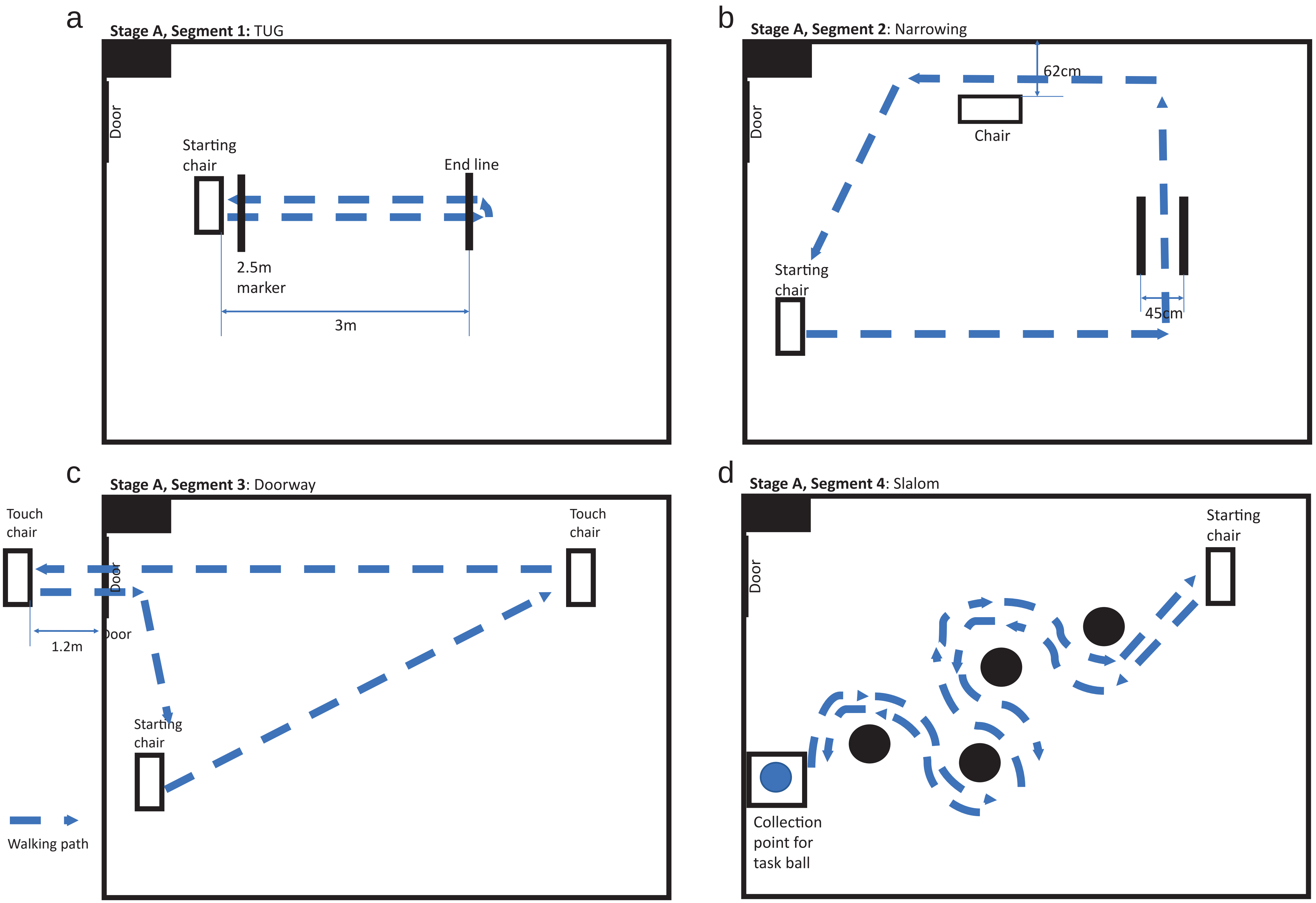

Supplementary Figure 2. Design of walking circuits during study stage A. a. Segment 1, timed up-and-go test. **b.** Segment 2, narrow restriction. **c.** Segment 3, passing through an open doorway. **d.** Segment 4, slalom around cones. **e.** Segemnt 5, step over and multitasking.

e

Stage A, Segment 5: Step over and multitasking

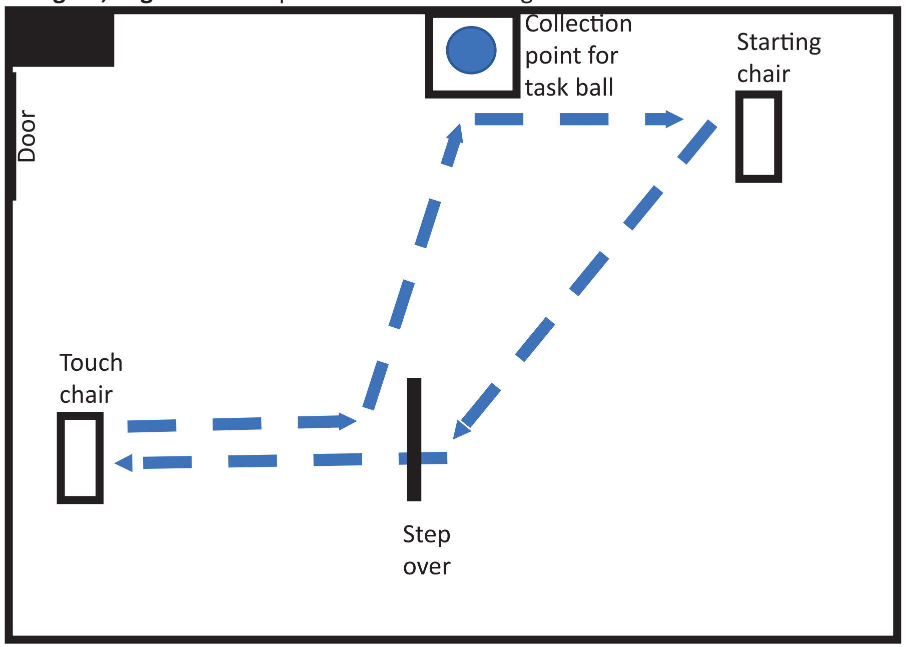

Supplementary Figure 2. Design of walking circuits during study stage A. a. Segment 1, timed up-and-go test. **b.** Segment 2, narrow restriction. **c.** Segment 3, passing through an open doorway. **d.** Segment 4, slalom around cones. e. Segemnt 5, step over and multitasking.

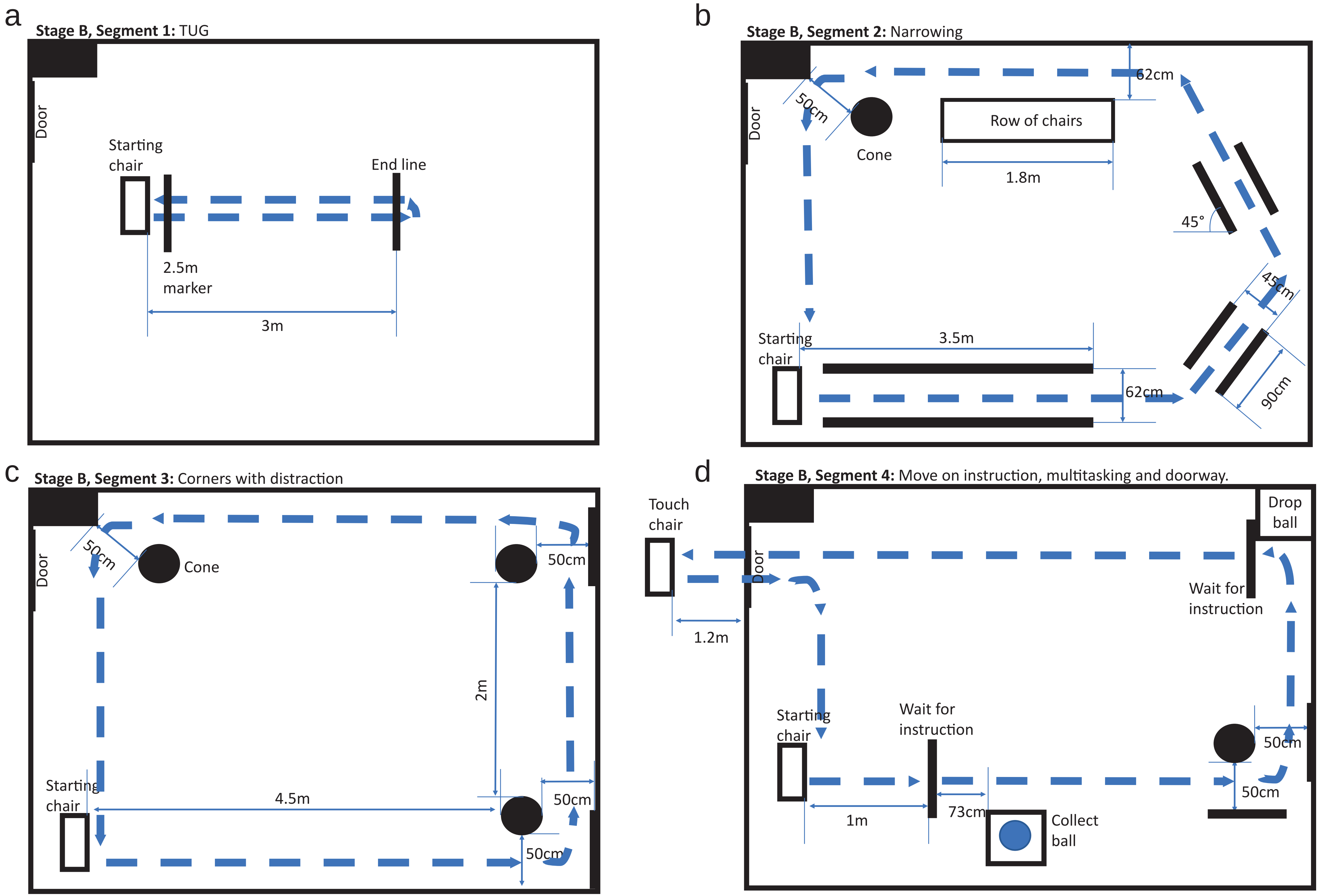

Supplementary Figure 3. Design of walking circuits during study stage B. a. Segment 1, timed up-and-go test. **b.** Segment 2, narrow restrictions. **c.** Segment 3, walking and turning with distraction. **d.** Segment 4, Move on instruction, multitasking and passing open doorway.

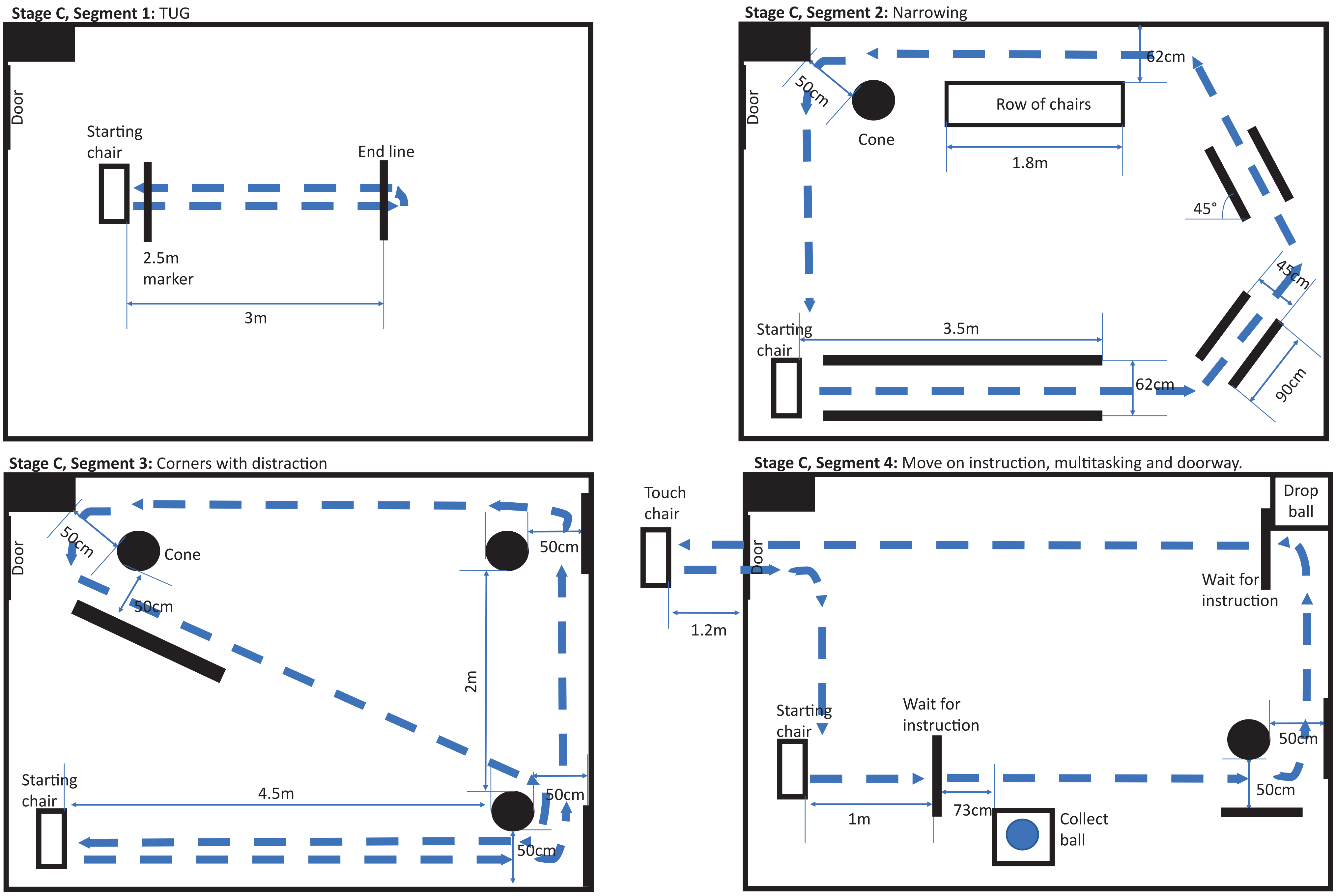

Supplementary Figure 4. Design of walking circuits during study stage C. a. Segment 1, timed up-and-go test. **b.** Segment 2, narrow restrictions. **c.** Segment 3, walking and tight turns with distraction. **d.** Segment 4, Move on instruction, multitasking and passing open doorway.

Supplementary Methods: GaitAnalyst Video analysis program

GaitAnalyst is custom video gait analysis software coded in Python3.3, to enable individual steps and other events/gait features to be marked and time-stamped against the video recording using keyboard strokes during video playback, which could be run at half and quarter speeds to increase accuracy of feature analysis. A screen shot of the GaitAnalyst interface is shown below. We provide open access to the software here. Video recordings were fragmented into individual files for each circuit for each participant (4 files per 1hours session per participant) and sound removed. Video recordings were viewed in random order and scored independently by 3 observers who had been trained to recognise the relevant gait features and to use the video analysis software. Each observer could recognise the no-device (ND) group from the video, however they were blinded with regard to the no-cue (NC), responsive cue (RC) and continuous cue (CC) interventions. Therefore, during statistical analysis, the active cueing groups (RC and CC) were compared with the device no-cue (NC) control, to avoid the potential for observer bias.

The GaitVideoAnalysis program is programmed in Python3.3 and is now available for open access. The program interface is present in Fig. 3.

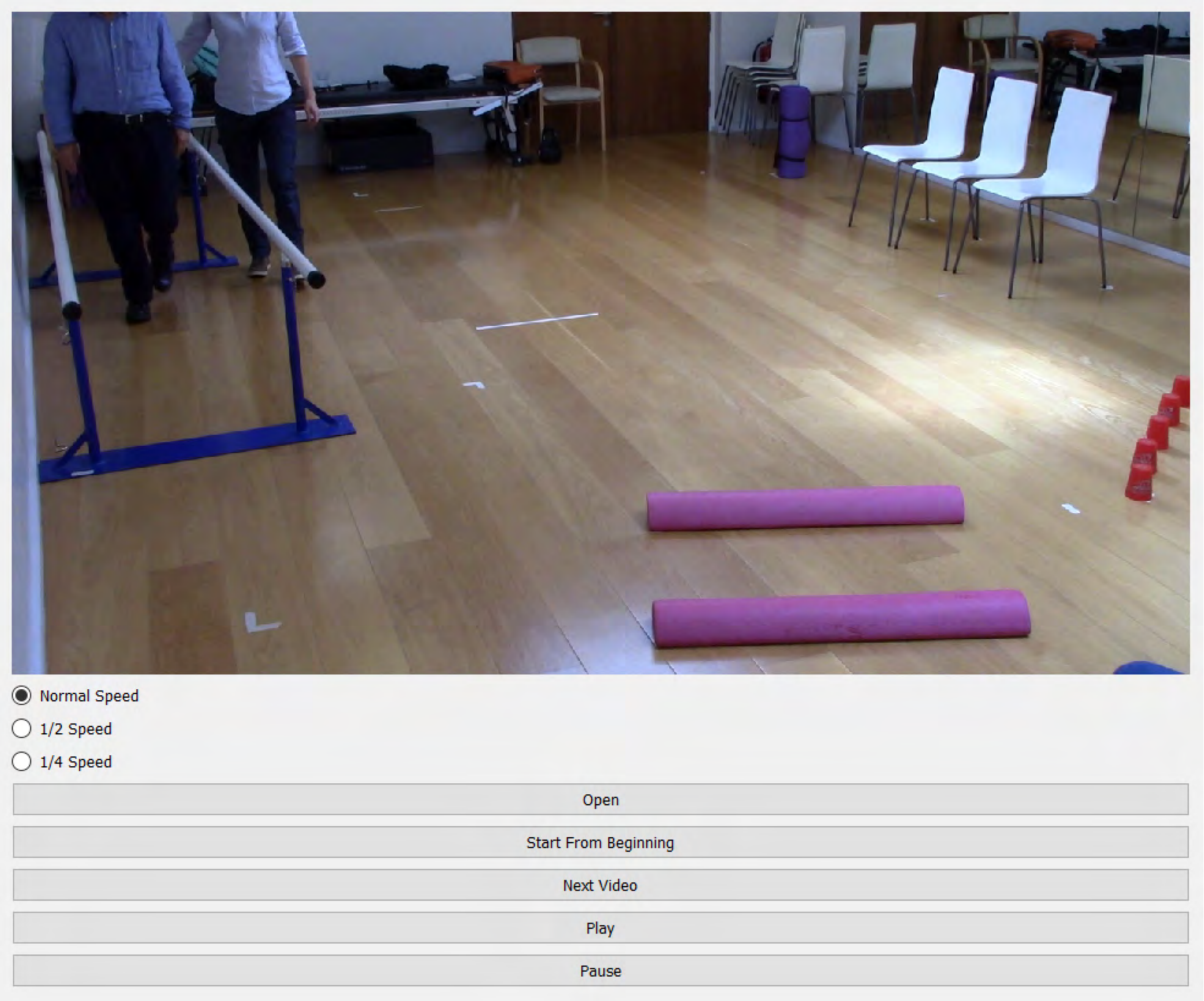

The GaitAnalyst interface

The main features of the program are:

1) Step timing: while the video is playing, observers can label the left or right steps by pressing the LEFT or RIGHT keys on the keyboard. The program records automatically the time (in milliseconds), the key being pressed and generates a report containing such information (screen shot of report below). This enables calculation of step frequency and symmetry.
2) FOG: the start of a gait freezing event is labelled/recording by pressing the UP key. The end of a gait freezing event is recorded as the start of normal walking/stepping (i.e., the subsequent left or right step).
3) Invalid time labels: if the observer cannot determine the walking characteristics of the participant due an obscured view or other issue, the observer can press the DOWN key to record this. The duration from this DOWN stroke to a valid event label (i.e., step and/or FOG keystroke) is omitted from the analysis.
4) Slow-motion play back: the program allows video playback in normal (actual recorded speed) and slow-motion modes (half and quarter speeds). This feature is particularly useful for accurately labelling left and right stepping and studying shuffling events.

```
File   Edit   Format   View   Help
L 00:00:00:864
R 00:00:01:296
L 00:00:01:662
R 00:00:01:994
L 00:00:02:427
R 00:00:02:792
L 00:00:03:191
R 00:00:03:524
L 00:00:03:856
R 00:00:04:189
L 00:00:04:521
R 00:00:04:887
L 00:00:05:352
R 00:00:05:784
L 00:00:06:017
R 00:00:06:217
L 00:00:06:516
R 00:00:06:815
L 00:00:07:148
R 00:00:07:414
L 00:00:07:945
U 00:00:08:178
R 00:00:13:996
L 00:00:14:362
```

A sample GaitAnalyst report.

Link to GaitAnalyst code: https://github.com/dongchengli940126/KeyPressedTimeVideoRecord

Supplementary Table 1: Pre-study Questionnaire (all participants)

Participant ID	Age	Gender	Have you been diagnosed with PD by a neurologist?	How long have you been diagnosed with PD (years)?	Do you suffer from gait difficulties			Do you sometimes require assisstance for standing and walking?	Current level of physical activities	In which situation are you more prone to suffering from gait difficulties	How do you re-initiate walking when suffering from gait difficulties
					Festination	Freezing	Other gait problems				
P0	75-79	M	Yes	5-9	Yes	Yes<br>Daily	Falls	Yes<br>Walking stick	High<br>Regular specialised physical activities	Anxiety inducing situations<br>Medication off<br>Manoeuver	Internal cueing
P1	75-79	M	Yes	5-9	Yes	Yes<br>Daily		Yes	Low<br>Some in-house activities	Multi-tasking<br>Fatigue	Internal cueing<br>Visual cueing
P2	75-79	M	Yes	10-14	No	Yes<br>Daily		Yes<br>Walking stick	Moderate<br>Regular in-house activities	Crowded situations<br>Manoeuver	Internal cueing<br>Weight shifting
P3	75-79	M	Yes	10-14	Yes	Yes<br>Daily		Yes<br>Support from others	High<br>Regular specialised physical activities	Crowded situations<br>Manoeuver<br>Anxiety inducing situations<br>Multi-tasking	Pause and restart<br>Auditory cueing
P4	75-79	M	Yes	5-9	Yes	Yes<br>Daily		No	Moderate	Fatigue	Self encouragement<br>Internal cueing
P5	65-69	M	Yes	10-14		Yes<br>Daily	Falls	Yes<br>Walking stick	Moderate<br>Regular in-house activities	Crowded situations<br>Anxiety inducing situations	Internal cueing<br>Auditory cueing
P6	75-79	M	Yes	10-14	Yes	Yes<br>Daily		Yes	Moderate<br>Regular in-house activities	Anxiety inducing situations	Pause and restart
P7	70-74	M	Yes	5-9		Yes<br>Daily		No	Moderate<br>Regular in-house activities	Initiating movement	Weight shifting
P8	80-84	M	Yes	5-9	Yes	Yes<br>Daily		Yes<br>Walking stick	Moderate	Crowded situations	Pause and restart
P9	75-79	M	Yes	10-14	Yes	Yes<br>Daily	Falls	Yes<br>Walker and wheelchair	Low<br>Some in-house activities	Initiating movement	No good method identified
P10	80-84	M	Yes	20-24		Yes<br>Daily	Falls	Yes<br>Walker	Moderate<br>Regular in-house activities	Crowded situations<br>Initiating movement	No good method identified
P11	65-69	F	Yes	5-9		Yes<br>Daily		Yes	Moderate<br>Regular in-house activities	Crowded situations<br>Anxiety inducing situations	Self encouragement<br>Self cueing
P12	80-84	F	Yes	10-14		Yes<br>Daily		Yes<br>Walker and wheelchair	High<br>Intense in-house activities	Random	Pause and restart
P13	75-79	F	Yes	5-9	Yes	Yes<br>Daily		Yes<br>Walking stick	Moderate<br>Regular in-house activities	All situations	Pause and restart
P14	65-69	M	Yes	15-19	Yes	Yes<br>Daily	Falls	Yes	Moderate<br>Regular in-house activities	Crowded situations	Self cueing
P15	65-69	M	Yes	10-14		Yes<br>Daily	Slow movement	Yes<br>Walking stick	Low<br>Some in-house activities	Initiating movement	Pause and restart
P16	60-64	M	Yes	5-9		Yes<br>Daily		No			Posture correction

Supplementary Table 2: pre-study FoG Questionnaire

Answer scoring:	1. During your worst state, do you walk:	2. Are your gait difficulties affecting your daily activities and independence:	3. Do you feel that your feet get glued to the floor while walking, making a turn or when trying to initiate walking (freezing)?	4. How long is your longest freezing episode?	5. How long is your typical start hesitation episode (freezing when initiating the first step)?	6. How long is your typical turning hesitation (freezing when turning)?
0	Normally	Not at all	Never	Never happened	None	None
1	Almost normally – somewhat slow	Mildly	Very rarely – about once a month	1-2s	Takes longer than 1s to start walking	Resume turning in 1-2 s
2	Slow but fully independent	Moderately	Rarely – about once a week	3-10s	Takes longer than 3s to start walking	Resume turning in 3-10 s
3	Need assistance or walking aid	Severely	Often – about once a day	11-30s	Takes longer than 10s to start walking	Resume turning in 11-30 s
4	Unable to walk	Unable to walk	Always – whenever walking	Unable to walk for more than 30s	Takes longer than 30s to start walking	Unable to resume turning for more than 30 s

Participant ID	Total FoG score (24 max)	1. During your worst state, do you walk:	2. Are your gait difficulties affecting your daily activities and independence:	3. Do you feel that your feet get glued to the floor while walking, making a turn or when trying to initiate walking (freezing)?	4. How long is your longest freezing episode?	5. How long is your typical start hesitation episode (freezing when initiating the first step)?	6. How long is your typical turning hesitation (freezing when turning)?
P0	**9.0**	2	1		2	2	2
P1	**12.5**	2.5	2	3.5	1	1.5	2
P2	**14.0**	3	2	3	2	2	2
P3	**10.0**	2	2	2	2	1	1
P4	**FoG questionnaire not returned**	n/a	n/a	n/a	n/a	n/a	n/a
P5	**18.0**	4	2	3	4	3	2
P6	**FoG questionnaire not returned**	n/a	n/a	n/a	n/a	n/a	n/a
P7	**12.0**	2	1	3	3	3	0
P8	**FoG questionnaire not returned**	n/a	n/a	n/a	n/a	n/a	n/a
P9	**21.0**	3	3	4	4	4	3
P10	**FoG questionnaire not returned**	n/a	n/a	n/a	n/a	n/a	n/a
P11	**15.0**	4	1.5	3.5	4	1	1
P12	**FoG questionnaire not returned**	n/a	n/a	n/a	n/a	n/a	n/a
P13	**12.0**	3	3	4			2
P14	**21.0**	4	2.5	3.5	4	4	3
P15	**21.0**	4	3	3	4	3	4
P16	**15.5**	4	1	3.5	3	2	2

Supplementary Table 3: post-study Questionnaire - Stage A

Participant ID	Did you feel that the device helped improve your walking?	Do you feel more confident when walking with the device?	Did wearing the device feel comfortable? How could it be improved?	How did you find the vibration strength?	How do you find the vibration duration?	How do you feel about the cueing frequency?	Would you like to use a device like GaitThaw in your daily life?	Any other comments
P0	Not sure. I was aware of my stepping.	Marginally.	Yes.	Should be stronger.	Should be shorter and sharper.	Close to my walking pace.	Yes, but would need to be silent.	No.
P1	Not sure.	Not sure.	Yes.	Should be a little stronger.	Should be longer (may help me react).	Close to my walking pace, but reaction time is hard.	Yes.	No.
P2	Marginally.	No.	Yes.	At right level.	At right level.	Too fast during turning and too slow for straight line.	Yes, would like to try.	Device is very light, I don't notice it.
P3	No (no freezing in study).	Not sure.	Yes.	Should be stronger.	At right level.	Faster than my walking pace.		Freezes in time pressured situations such as boarding a bus.
P4	Yes and no: I didn't freeze. Would like to try on a bad day.	Marginal improvement in confidence.	Yes.	At the right level for a good day. Should be stronger for a bad day.	At right level.	Slower than my walking pace.	Yes. I would like to test on a bad day at home.	Interesting and useful.
P5	Yes.	Yes.	Yes.	Should be stronger.	At right level.	Needs to be tuned into my walking pace in different environment.	Yes "the best thing I have tried so far."	Tests could be more challenging. Device needs to be quieter
P6	Not sure.	Yes.	Yes.	At right level.	At right level.	Faster than my walking pace	Yes, would would like to try.	
P7	Not sure.	No.	Yes.	Should be stronger.	At right level.	Slower than my walking pace.	Not sure.	Well organised study.
P8	No.	No.	Yes.	At right level.	At right level.	Slower than my walking pace.	Not sure - no freezing today	No
P9	Yes. Provided inputs to move.	Yes.	Yes.	Should be stronger.	At right level.	Close to my walking pace.	Yes.	
P10	Yes. Without a doubt.	Not sure.	Yes.	Should be stronger.	At right level.	Slower than my walking pace.	Yes.	
P11	Yes. Comforting rhythm, soothing, sound was also helpful, rhythmic (continuous) cue made me more steady.	Yes. It was comforting.	Yes.	Should be stronger.	At right level.	Faster than my walking pace in countryside, close to my "shopping pace".	Yes. I would give it a try.	Friendly team. Longer distance walking would be good.

Supplementary Table 4: post-study Questionnaire - Stage B

Participant ID	Did GaitThaw device improve your walking?	Do you feel more confident when walking wearing the device	Do you feel comfortable wearing the device? How could it be improved?	How do you feel about vibration strength	How do you feel about vibration duration	How do you feel about cueing frequency	Would you use GaitThaw device in daily life	Any other comments
P0	Yes. It helped me concentrate (therefore improved walking).	Yes, marginally more confident.	Yes, it was comfortable.	Should be weaker (but only to reduce noise). At the right level (but could be slightly softer).	At right level.	Right level, possibly a little slow.	I could do. It may reduce my fall risk. I like that both hands are free.	The gym environment may not represent my everyday environment (e.g. physio effect).
P1	Not sure.	Not sure.	Yes it was comfortable.	At right level.	At right level.	Close to my walking pace	Yes, I would consider using this.	
P4	No.	No.	Yes it was comfortable	At right level.	At right level.	At right level.	Would certainly like to try it: especially when freezing but not fatigued, or when freezing in everyday environment. Unfamiliar environment is often the trigger. Too little or too much space (fear of falling).	No freezing in 1st/2nd circuit. Fatigued in 3rd and 4th circuit – freezing started. Didn't seem to help with walking when fatigued. Would like to try when freezing but not fatigued. Often freezes when facing oncoming pedestrians in crowded environment (e.g. airport).
P5	Yes. Fewer stumbles with cueing.	Not sure	It was too comfortable, too warm and fuzzy. Stimulus needs to be sharper.	Should be stronger, less diffuse and sharper.	Should be shorter: they tended to shade into each other.	At the right level. Hard to judge as stimulus blurred into each other.	Yes I would like to use – especially if modified based on above	I would really like to have a manual trigger for the continuous cue
P7	Yes: it helped me concentrate more on my walking.	Not sure	Yes it was comfortable	At right level	At right level		(Yes) I Certainly wouldn't mind trying it	I had cramps the last few days/nights. The continuous cueing had a soothing effect.
P11	Yes.	Yes. I felt I didn't freeze as much. Walking felt more measured.	Yes it was comfortable.	Should be stronger on the right (bad) side. Is at the right level on the left (good) side.	At right level.	At right level.	I would like to try it at home (especially in the garden). Would be very useful after standing for long periods (I struggle to get started – stutter).	
P12	Yes. First continuous cue did help.	No.	Yes it was comfortable.	At right level.	Should be longer perhaps.	At right level.	Yes, I could use it at home.	
P13	Yes. Felt I could move my legs.	Yes. I liked having it along with my crutch.	Yes it was comfortable.	At right level.	At right level.	At right level.	Yes	

Supplementary Table 5: post-study Questionnaire - Stage C

Participant ID	Did you feel that the device helped improve your walking?	Do you feel more confident when walking with the device?	Did wearing the device feel comfortable? How could it be improved?	How did you find the vibration strength?	How do you find the vibration duration?	How do you feel about the cueing frequency?	Would you like to use a device like GaitThaw in your daily life?	Any other comments
P0	Yes, pacing (continuous) cue helped me slow down. Helped me concentrate: I'm easily distracted and often fall, so this helped me concentrate	Not sure: when concentrating I'm always a bit uncertain; when not concentrating my fall risk increase	Yes, it was comfortable	At the right level.	At the right level.	At the right level.	Yes I would like to try at home: that's where I fall most.	Needs to be quieter.
P1	Yes, it helped me focus and avoid distractions	Yes. By focussing on the cue I felt more stable (less likely to fall).	Not uncomfortable.	At the right level (perhaps could be a little stronger)		At the right level.	Yes, I would.	I felt fatigued during the final circuit: it was very tiring! I was able to get back into rhythm with the cue if I became out of sync.
P2	Yes, it helped on the long stretches (continuous cue). No, it didn't help on turns, but I was missing my usual contact points	Not sure. Lack of contact points reduced confidence	Yes, it was comfortable	At the right level.	At the right level.	At the right level.	Don't know. Would like to try a bit more in a familiar setting (at home).	Usually I would use a contact point (even as small as a twig) at each turn to maintain stability and help navigate the turn. Today I found it more challenigng on the corners/turns without a physical contact point.
P3	No.	Yes. Good having a rhythm to walk to.	Yes it was comfortable worn against skin.	At the right level.	At the right level.	Slower than my walking pace.	Yes I would like to try at home.	I have felt quite hesitant today (in a doorway).
P7	Yes: Rhythmic (continuous) cue helped me slow my pace and focus. No: responsive cue didn't help as it wasn't needed (due to lack of freezing).	No	Yes, it was comfortable	At the right level.	At the right level.	At the right level (I was conciously slowing my pace)	Would like to try when freezing at home. I seem not to freeze in this study clinic.	I seem not to freeze in this study clinic: but I do at home. Common triggers are walking short distances after a complex task (e.g. watering garden after filling can). I seem to either freeze OR have tremor on any given day/time. I find the freezing far more disruptive than tremor. Very hard to do two things at once (motor dual task): has come on in last 2 weeks
P9	Yes.	Yes. It was a comfort being able to feel the cue.	Yes, it was comfortable.	At the right level.	At the right level.	Slower than my walking pace.	Yes I would.	The study has been very good.
P11	Yes: it made me slow down and walk with a moderate speed.	Yes: probably. Made me think about my walking. It may have prevented festination.	Yes, it was comfortable.	At the right level.	At the right level.	Slower than my walking pace.	Yes I would.	Initially I was distracted by the device as I couldn't match the rhythm. Once I got the rhythm I was fine. It would be good to add music, or a rock and roll beat.
P13	Yes, I felt I was walking more positively when it was cueing.	Yes.	Yes, it was comfortable.	At the right level.	At the right level.	At the right level.	Yes I would.	
P14	Yes, both cue types (helped)	Yes, both cue types (improved confidence).	Yes, it was comfortable.	At the right level.	At the right level.	Slower than my walking pace. Nice pace for bimbling along, but for normal day to day would like a bit quicker,	Yes I would,	Responsive cue really changed my walking: it helped me initiate walking with my right or left leg; usually I lead with my left. Rhythmic (continuous) cue was really good. Felt more relaxed and helped me multitask. It made me slow down giving me more time to judge where I'm going and better navigate corners. It prevented stride length from shortening, both in straight lines and around corners. Would be nice if the pace could be adjusted for leisurely vs purposeful walking.
P15	Yes.	Yes.	Yes, it was comfortable.	At the right level.	At the right level.	At the right level.	Yes, if I can have some more time with the device.	
P16	Yes: I felt that the rhythmic (continuous) cue helped, but I didn't freeze enough to test the responsive cue.	Yes: May help me walk further.	Yes, it was comfortable.	At the right level	Should be shorter (short and sharp).	At the right level: fine inside, but if I was outside I would want to go faster.	Yes, definitely.	It would be good to make the device quieter. I would like the option of a manual trigger as well as the automatic function.
